# Glycogen Phosphorylase Inhibitor N-(3,5-Dimethyl-Benzoyl)-N’-(β-D-Glucopyranosyl)Urea Improves Glucose Tolerance under Normoglycemic and Diabetic Conditions and Rearranges Hepatic Metabolism

**DOI:** 10.1371/journal.pone.0069420

**Published:** 2013-07-25

**Authors:** Lilla Nagy, Tibor Docsa, Magdolna Szántó, Attila Brunyánszki, Csaba Hegedűs, Judit Márton, Bálint Kónya, László Virág, László Somsák, Pál Gergely, Péter Bai

**Affiliations:** 1 Department of Medical Chemistry, University of Debrecen Medical and Health Science Center, Debrecen, Hungary; 2 Cell Biology and Signaling Research Group of the Hungarian Academy of Sciences, Debrecen, Hungary; 3 Department of Organic Chemistry, University of Debrecen, Debrecen, Hungary; Hungarian Academy of Sciences, Hungary

## Abstract

Glycogen phosphorylase (GP) catalyzes the breakdown of glycogen and largely contributes to hepatic glucose production making GP inhibition an attractive target to modulate glucose levels in diabetes. Hereby we present the metabolic effects of a novel, potent, glucose-based GP inhibitor (KB228) tested *in vitro* and *in vivo* under normoglycemic and diabetic conditions. KB228 administration enhanced glucose sensitivity in chow-fed and obese, diabetic mice that was a result of higher hepatic glucose uptake. Besides improved glucose sensitivity, we have observed further unexpected metabolic rearrangements. KB228 administration increased oxygen consumption that was probably due to the overexpression of uncoupling protein-2 (UCP2) that was observed in animal and cellular models. Furthermore, KB228 treatment induced mammalian target of rapamycin complex 2 (mTORC2) in mice. Our data demonstrate that glucose based GP inhibitors are capable of reducing glucose levels in mice under normo and hyperglycemic conditions. Moreover, these GP inhibitors induce accommodation in addition to GP inhibition - such as enhanced mitochondrial oxidation and mTORC2 signaling – to cope with the glucose influx and increased glycogen deposition in the cells, however the molecular mechanism of accommodation is unexplored.

## Introduction

Glycogen content of tissues and cells depend on the concerted regulation of glycogen synthesis by glycogen synthase (GS) and glycogen breakdown by glycogen phosphorylase (GP) through an intricate network of signal transduction pathways related to hormonal signaling [1]. These signal transduction pathways, converging on GS and GP, exert their regulatory activity through the posttranslational modification of these enzymes to meet the energy demands of the organism [2]–[4].

GP activity is crucial in fine tuning hepatic glycogen content and hepatic glucose homeostasis [5], [6]. Glycogen breakdown by GP is associated with fasting responses that lead to enhanced hepatic glucose production (HGP) [7] that is reduced by GP inhibition. Moreover, GP inhibition enhances glycogen build-up in skeletal muscle and liver enhancing glucose uptake that contributes to glucose clearance from blood [8], [9]. Inhibition of HGP and induction of glucose uptake together reduce blood glucose that makes GP a promising pharmaceutical target to manage serum glucose levels.

GP is a homodimeric enzyme existing in a phosphorylated (GPa) and an unphosphorylated form (GPb) [10]. Phosphorylase kinase phosphorylates GPb turning it to GPa, the active form [10]. Effectors influence GP activity by switching between the tense (T, less active) and relaxed (R, more active) states of both GPa and GPb. There are several effector binding sites on GP: the active site, the allosteric (AMP binding) site, the new allosteric (indole-carboxamide binding) site, the inhibitor (purin binding) site and the storage site. [11]. GP has three isoforms named after the tissues where it is dominantly expressed: liver (pygl), brain (pygb) and muscle (pygm). Most GP inhibitors (GPi-s) are unselective and inhibit all isoforms [10], [11].

It is important to note that glucose is considered as a physiological regulator of GP [12]. However, glucose 6-phosphate exerts a similar effect on GP as glucose, although glucose and glucose 6-phosphate bind to different sites [13] and their binding converts GPa to the T conformation making it more prone to dephosphorylation [13].

Research efforts have identified an ample number of structurally different, potent GPi-s (reviewed in [7], [14]). Genetic or pharmacological inhibition of GP activity ameliorates glucose tolerance supporting the possible applicability of GP inhibition in the management of glucose handling disorders in diabetes [8], [9], [15]–[18]. Indeed, a GP inhibitor, CP-316819 (Ingliforib), in clinical study was able to reduce glucagon-induced hyperglycemia [11].

Our research group has been involved in the design of glucose-derived and other GPi-s [19], [20]. In the current study we have characterized the metabolic effects of a novel glucose-based GPi N-(3,5-dimethyl-benzoyl)-N’-(β-D-glucopyranosyl)urea (**KB228**) in control, and diabetic mice and in cellular models.

## Materials and Methods

### Chemicals

Unless otherwise stated, all chemicals were from *Sigma-Aldrich* (St. Louis, MO, USA).

Glycogen phosphorylase inhibitors TH (D-glucopyranosylidene-spiro-thiohydantoin) [9], NV50 (*N*-(β-D-glucopyranosyl)-*N’*-(4-nitrobenzoyl) urea) [21] and NV76 (*N*-(β-D-glucopyranosyl)-*N’*-(2-naphthoyl) urea) [14], [22] were synthesized in the laboratory of Dr. László Somsák and were described in the literature indicated.

### Preparation of N-(3,5-dimethyl-benzoyl)-N’-(β-D-glucopyranosyl)urea

Preparation of acyl-isocyanates was adapted from literature [23]: Oxalylchloride (1.1 equivalent) was added to a suspension of 3,5-dimethyl-benzamide **2** [24] (200 mg, 1.341 mmol) in anhydrous 1,2-dichloroethane (15 mL) and the mixture was heated at reflux temperature for 1 day. The volatiles were distilled off under diminished pressure and toluene (2 × 5 mL) was evaporated from the residue to remove the rest of oxalylchloride. The crude acyl-isocyanate **3** obtained in this way was mixed with a solution of β-D-glucopyranosylammonium carbamate [25] (**1**, 320 mg, 1.341 mmol, 1 equivalent) in anhydrous pyridine (45 mL) and the mixture was stirred at room temperature for 4 days. Pyridine was distilled off under diminished pressure and evaporation of toluene (2 × 30 mL) removed traces of pyridine. The crude product was purified by silica gel column chromatography (CHCl_3_-MeOH, 7∶1) to give the target compound **4**.

Yield: 214 mg (45%), yellow syrup. R_f_ = 0.21 (CHCl_3_-MeOH, 7∶1) [α]_D_ +7.1 (c = 0.310, DMSO) ^1^H NMR (DMSO-d_6_, 360 MHz) δ (ppm) 10.70 (s, 1H, N*H*), 9.09 (d, 1H, *J* = 9.1 Hz, N*H*), 7.59 (s, 2H, Ar*H*), 7.26 (s, 1H, Ar*H*), 5.25 (d, 1H, *J* = 5.4 Hz, OH), 5.03 (d, 1H, *J* = 4.6 Hz, OH), 4.94 (d, 1H, *J* = 4.9 Hz, OH), 4.81 (t, 1H, *J* = 9.0, 9.0 Hz, H-1), 4.53 (pseudo t, 1H, *J* = 9.2, 8.9 Hz, OH), 3.65 (dd, 1H, *J* = 12.1, 2.3 Hz, H-6a), 3.42 (dd, 1H, *J* = 12.1, 3.9 Hz, H-6b), 3.25-3.00 (m, 4H, H-2, H-3, H-4, H-5), 2.32 (s, 6H, 2xC*H*_3_).^13^C NMR (DMSO-d_6_, 90 MHz) δ (ppm) 169.2 (NH*C*OAr), 156.3 (NH*C*ONH), 133.9, 129.1, 126.7, 122.9 (Ar), 82.1 (C-1), 78.9, 78.1, 75.1, 71.7 (C-2–C-5), 63.0 (C-6), 22.3 (*C*H_3_). Anal. Calcd for C_16_H_22_N_2_O_7_ (354.36): C, 54.23; H, 6.26; N, 7.91; Found: C, 54.37; H, 6.15; N, 8.03.

### Biochemical Measurements

GP kinetic studies were performed as described in [26]. Determination of glycogen was as in [9].

### Animal Studies

All animal experiments were carried out according to the national, EU and NIH ethical guidelines and were authorized by the Institutional Animal Care and Use Committee at the University of Debrecen (7/2010 DE MÁB). C57/Bl6J male mice (*Charles River*, Wilmington, MA, USA) had *ad libitum* access to water and chow (10 kcal% of fat) (SAFE, Augy, France) or hypercaloric high-fat diet (HFD, 60 kcal% of fat) (*Research Diets, Inc.*, New Brunswick, NJ, USA), and were kept in a 12 hr dark/light cycle (light 7 a.m. –7 p.m., night 7 p.m. –7 a.m.). All measurements took place 2 hours after injecting 90 mg/kg KB228 in a single intraperitoneal (i.p.) bolus unless otherwise stated. Intraperitoneal glucose tolerance test (ipGTT) and intraperitoneal insulin tolerance test (ipITT) was described in [27].

### Indirect Calorimetry

Indirect calorimetry experiments were performed in a CLAMS system (*Columbus Instruments*, Columbus, OH, USA). Mice were habituated to the new environment of the cages for 24 hours. Then at 8 a.m. mice received a bolus i.p. injection of KB228 (90 mg/kg) or vehicle then were returned to the measurement cages and for the following six hours oxygen consumption and carbon dioxide release was recorded.

### Glucose Uptake Experiments

In glucose uptake experiments mice were injected with 120 µCi/kg ^14^C-2-deoxyglucose and 20 U/kg insulin through the jugular vein under halothane anaesthesia. The incision above the vein was closed with a suture. Blood glucose levels were monitored at the time of the suture, 15 and 30 minutes post intervention. Mice were sacrificed by cervical dislocation 30 minutes post intervention and the indicated organs were removed. Carefully weighed pieces of these tissues were solubilised in 0.5 ml 1 M NaOH at 70°C for 60 minutes. Lysates were mixed with Aqualight HIBEX (*Personal Life Sciences*) scintillation liquid and were measured in a Wallac scintillation counter (*Perkin Elmer*, Waltham, MA, USA).

### Cell Culture

HepG2 human hepatocarcinoma cells and C2C12 myoblasts were obtained from ATCC (Manassas, VA, USA) and were cultured in DMEM, 10% FCS, 1 g/L or 4.5 g/L glucose, as indicated. C2C12 cells were differentiated in DMEM, 2% horse serum 1 g/L glucose for 2 days as described in [27], [28].

### Cellular Oximetry

*O*xygen *c*onsumption *r*ate (OCR) of HepG2 cells were measured using an XF96 oxymeter (Seahorse Biosciences, North Billerica, MA, USA) similarly to [29]. Briefly, HepG2 cells were seeded in 96-well assay plates. After recording the baseline OCR cells received a single bolus dose of 3 µM KB228 or other GPi-s, as indicated. Then, OCR was recorded every hour to follow the effects of GPi-s. Final reading took place at 8 hours post-treatment. OCR was normalized to protein content and normalized readings were displayed.

### cDNA Preparation, qPCR

Total RNA preparation, reverse transcription, and RT-qPCR were performed as in [30]. Expression was normalized to the geometric mean of three control genes (β-actin, cyclophylin, 36B4). Primers are summarized in Table 1.

**Table 1:** Primers used in RT-qPCR reactions.

Gene		Forward primer	Reverse primer
*β-actin*	Human	5′-GACCCAGATCATGTTTGAGACC-3′	5′-CATCACGATGCCAGTGGTAC-3′
	Murine	5′-TGGAGAGCACCAAGACAGACA-3′	5′-TGCCGGAGTCGACAATGAT-3′
*Cyclophylin*	Human	5′-GTCTCCTTTGAGCTGTTTGCAGAC-3′	5′-CTTGCCACCAGTGCCATTATG-3′
	Murine	5′-CAAGGTCATCCATGACAACTTTG-3′	5′GGCCATCCACAGTCTTCTGG-3′
*36B4*	Human	5′-CCATTGAAATCCTGAGTGATGTG-3′	5′-GTCGAACACCTGCTGGATGAC-3′
	Murine	5′-AGATTCGGGATATGCTGTTGG-3′	5′-AAAGCCTGGAAGAAGGAGGTC-3′
*UCP2*	Human	5′-CTACAAGACCATTGCCCGGAG-3′	5′-ACAATGGCATTACGAGCAACA-3′
	Murine	5′-TGGCAGGTAGCACCACAGG-3′	5′-CATCTGGTCTTGCAGCAACTCT-3′
*PYGL*	Human	5′-GTGCCCCAAGAGGGTATATTAC-3′	5′-AAGAAGCAGGCAGCAAGTCTC-3′
	Murine	5′-CCCCGTGCCTGGATATATGA-3′	5′-TGTTTCAGCCGCAACTCCTT-3′
*PYGM*:	Human	5′-GGACCCCAAGAGGATCTACTACC-3′	5′-CCTCGTCACAGGCATTCTCTA-3′
	Murine	5′-CCCAAGAGGATCTACTACCTGTC-3′	5′-ACTCATAGCGGATCCCATAGC-3′
*PYGB*	Human	5′-AGCCATCTATCAGTTGGGGTTAG-3′	5′-TGCCAGCCATTGACAATCTTC-3′
	Murine	5′-CACTTATCAGTTGGGGTTGGAC-3′	5′-GCCAGTCATCAGCTTCTTCAAC-3′

### Protein Extraction and Western Blotting

Western blotting experiments were performed as described in [28]. Blots were probed with the following antibodies: UCP2 (*Abcam*, Cambridge, UK, 1∶1000), Akt-2 (*Cell Signalling*, Danvers, MA, USA, 1∶1000), phospho-Akt-2 (^473^Ser) (*Cell Signaling*, 1∶2000), GSK3β (*Sigma-Aldrich*, 1∶2000), phospho-GSK3β (^9/21^Ser) (*Cell Signaling*, 1∶1000) and actin (*Sigma-Aldrich*, 1∶10000).

### Statistical Analysis

Significance was determined using unpaired t-test for unequal sized samples where *p*<0.05 was considered as significant. Error bars represent SEM unless stated otherwise.

## Results

### Preparation of N-(3,5-dimethyl-benzoyl)-N’-(β-D-glucopyranosyl)urea (KB228)

**Figure 1:**
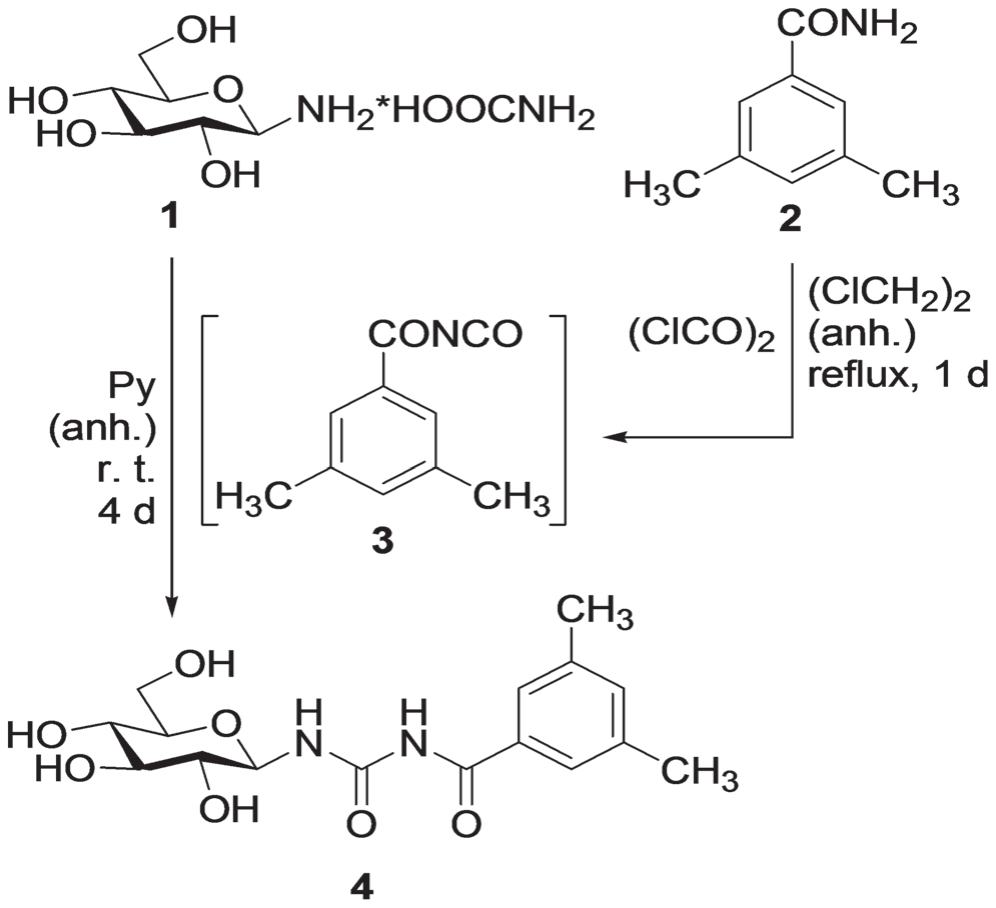
Synthesis of KB228. *N*-(3,5-dimethyl-benzoyl)-*N’*-(β-D-glucopyranosyl)urea (KB228), a GP inhibitor was prepared as described in the Materials and Methods section.

The glycogen phosphorylase inhibitor KB228 (*N*-(3,5-dimethyl-benzoyl)-*N’*-(β-D-glucopyranosyl)urea **4**) was prepared according to a previously published procedure [31] as shown in Fig. 1.: by treatment with oxalylchloride, 3,5-dimethyl-benzamide (**2**) was transformed into the corresponding acyl-isocyanate **3** which was reacted with β-D-glucopyranosylammonium carbamate (**1**) to yield the target compound **4**.

### Biochemical and Physiological Characterization of KB228

**Figure 2:**
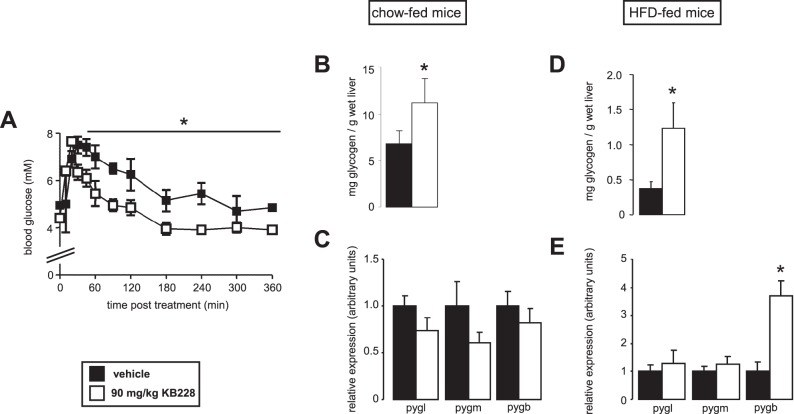
Characterization of the *in vivo* applicability of KB228. (A) C57/Bl6J male mice (n = 3/3, 3 months of age) were administered KB228, or vehicle (physiological saline, 1% DMSO) i.p., then blood glucose levels were determined using an Accu-Check glucometer (*Roche*). (B-C) Chow-fed C57/Bl6J male mice (n = 7/7, 6 months of age) were sacrificed 2 hours post treatment with KB228 (90 mg/kg) then (B) glycogen content and (C) the expression the liver, brain and muscle isoforms of GP (*pygl*, *pygb* and *pygm*, respectively) were determined using RT-qPCR. (D-E) HFD-fed C57/Bl6J male mice (n = 9/9, 6 months of age) were sacrificed 2 hours post treatment with KB228 (90 mg/kg) then (D) glycogen content and (E) the expression of the indicated genes were measured by RT-qPCR. * indicate statistically significant difference between vehicle and KB228-treated groups at p<0.05.

KB228 displayed mixed type inhibition on purified rabbit muscle glycogen phosphorylase and the K_i_ of the inhibitor was calculated to be 0.93±0.05 µM. Next, we set out to find an appropriate dose and administration of KB228 for *in vivo* studies. KB228 was administered to C57/Bl6J mice as a single i.p. injection in a 90 mg/kg dose (lower doses were ineffective – data not shown). KB228 treatment reduced blood glucose levels 30 minutes post treatment and the reduction was maintained for 6 hours (Fig. 2A) that coincided with an increment in hepatic glycogen content (Fig. 2B) without change in the expression of GP isoforms (Fig. 2C) suggesting that KB228 treatment was effective. We induced glucose intolerance and hampered insulin sensitivity (tested in ipGTT and ipITT, data not shown) by HFD feeding (3 months feeding). Significant increase in hepatic glycogen content confirmed the efficiency of GP inhibition (Fig. 2D). In that case we observed the induction of brain isotype GP (*pygb*), while other isoforms of GP did not change (Fig. 2E). The data validated the applicability of KB228 under normal and diabetic conditions in mice.

### Effect of KB228 on Energy Balance

**Figure 3:**
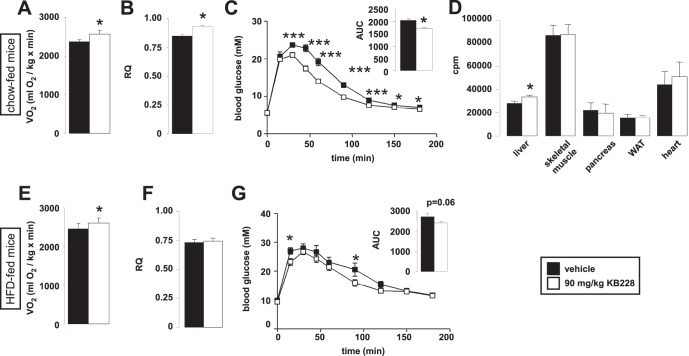
The impact of KB228 on *in vivo* glucose metabolism. (A-B) Chow-fed C57/Bl6J male mice (n = 7/7, 6 months of age) underwent vehicle or KB228 treatment, then (A) oxygen consumption and (B) RQ were determined in indirect calorimetry chambers. (C) The same cohorts of mice were subjected to an ipGTT test. (D) Chow-fed C57/Bl6J male mice (n = 4/4, 6 months of age) were subjected to a glucose uptake experiment as described in Materials and Methods. (E-F) HFD-fed C57/Bl6J male mice (n = 9/9, 6 months of age) underwent vehicle, or KB228 treatment, then (E) oxygen consumption and (F) RQ were determined in indirect calorimetry chambers. (G) The same cohorts of mice were subjected to an ipGTT test. * and *** indicate statistically significant difference between vehicle and KB228-treated groups at p<0.05, or p<0.001, respectively.

Given the long lasting effect of KB228, we performed indirect calorimetry experiments on C57/Bl6J mice that were on chow diet or HFD. Surprisingly, KB228 treatment enhanced oxygen consumption in chow-fed mice (Fig. 3A) suggesting an increased oxidative metabolism; and this result coincided with the higher respiratory quotient (RQ) found in chow-fed mice (Fig. 3B) indicative of higher glucose oxidation rates. In line with these data, KB228-treated mice displayed better glucose tolerance in ipGTT assays (Fig. 3C). Glucose uptake assays suggested that the main organ responsible for glucose excursion is the liver (Fig. 3D). KB228 treatment in HFD-fed animals had similar effects to chow-fed animals in terms of oxygen consumption and glucose tolerance (Fig. 3E–G). However, the improvement of their metabolic properties was less pronounced as compared to the chow-fed animals. There was only a slight increase in RQ on HFD instead of the significant enhancement on chow, furthermore ipGTT showed a weaker improvement (Fig. 3E–G), that was in line with the lower potency of KB228 to inhibit GP in hyperglycemic mice.

### KB228 Treatment Induces UCP2 Expression and mTORC2 Activity

**Figure 4:**
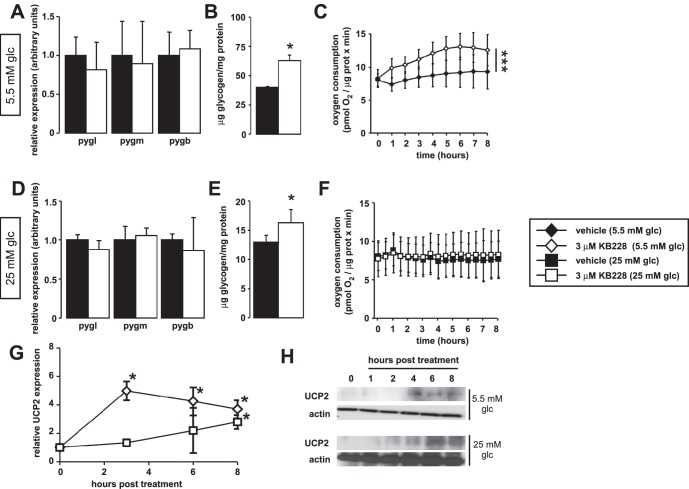
Metabolic effects of KB228 treatment in HepG2 cells. (A–C) In vehicle or KB228-treated HepG2 cells (n = 3/3) under normoglycemic conditions (A) the expression of GP isoforms, (B) glycogen content and (C) cellular oxygen consumption (OCR) were determined (n = 47/47). (D–F) In vehicle or KB228-treated HepG2 cells (n = 3/3) under hyperglycemic conditions (D) the expression of GP isoforms, (E) glycogen content and (F) cellular oxygen consumption (OCR) were determined (n = 47/47). (G–H) In vehicle, or KB228-treated HepG2 cells (n = 4/4) under normoglycemic and hyperglycemic conditions UCP2 (G) mRNA and (H) protein levels were determined by RT-qPCR and Western blotting, respectively. Error is given as SD throughout the figure. * and *** indicate statistically significant difference between vehicle and KB228-treated groups at p<0.05, or p<0.001, respectively.

Our *in vivo* data suggested metabolic rearrangements in liver; therefore, we explored the *in vitro* metabolic effects of KB228 on HepG2 cells under normoglycemic (5.5 mM glucose in the medium) and hyperglycemic conditions (25 mM glucose in the medium). The expression of GP isoforms were unaltered both under normo and hyperglycemia (Fig. 4A, D) and KB228 treatment induced glycogen build-up (Fig. 4B, E) suggesting effective GP inhibition in both conditions. The administration of KB228 to HepG2 cells enhanced mitochondrial oxidation under normoglycemia (Fig. 4C). Treatments under hyperglycemia exerted negligible effects on mitochondrial oxidation (Fig. 4F).

In RT-qPCR reactions we have observed that the expression of uncoupling protein-2 (UCP2) was enhanced upon KB228 treatment in HepG2 cells. UCP2 mRNA and protein levels were induced by KB228 in a time-dependent manner (Fig. 4G–H). Induction of UCP2 expression and protein levels were not as pronounced in hyperglycemic conditions as in normoglycemia (Fig. 4G–H). UCP2 is a likely candidate to explain enhanced catabolism upon KB228 treatment.

**Figure 5:**
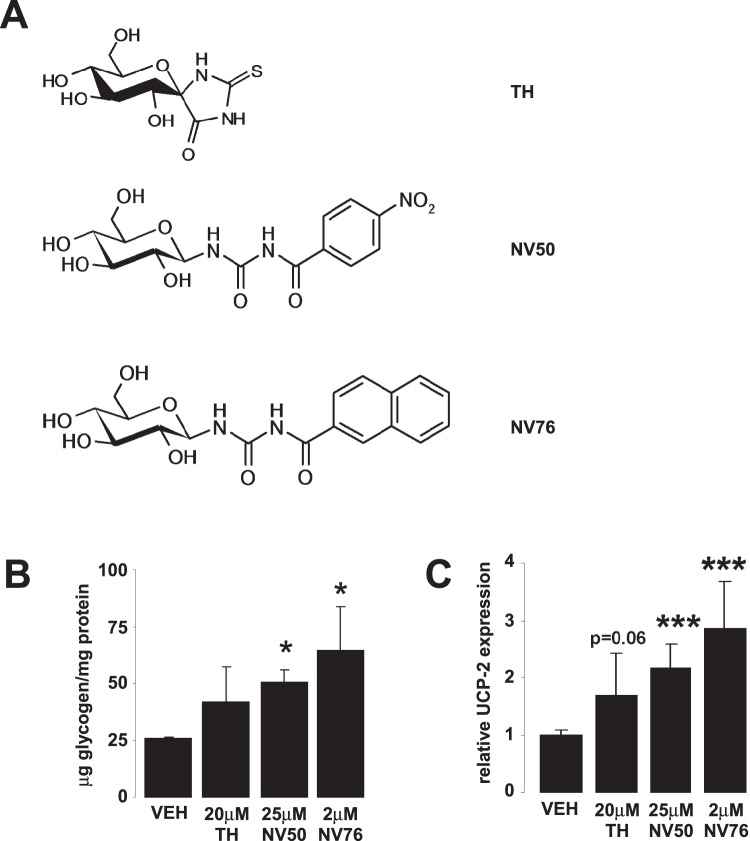
Other GPi-s also induce UCP2 expression in HepG2 cells. (A) Three GPi-s TH, NV50 and NV76 were tested on HepG2 cells. (B–C) HepG2 cells (n = 3) kept under normoglycemic conditions were treated with the inhibitors at the indicated concentrations for 8 hours, then (B) glycogen content and (C) UCP-2 expression was determined as described in Materials and Methods. Error is given as SD throughout the figure. * and *** indicate statistically significant difference between vehicle and GPi-treated groups at p<0.05, or p<0.001, respectively. VEH – vehicle, other abbreviations are in the text.

We tested other potent GPi-s (TH, K_i_ = 5.1 µM [9], NV50, K_i_ = 3 µM [21] and NV76, K_i_ = 0.47 µM [14], [22]) (Fig. 5A) on HepG2 cells cultured under normoglycemic conditions. These GPi-s efficiently inhibited GP as demonstrated by increases in cellular glycogen content (Fig. 5B). Furthermore, the treatment of cells with these GPi-s led to a ∼2 fold induction of UCP2 expression (Fig. 5C) similarly to KB228. These data demonstrate that the induction of UCP2 is not limited to KB228 only but can be elicited by other inhibitors too. Furthermore, potency of the drugs to induce UCP-2 expression correlated with their respective K_i_’s.

**Figure 6:**
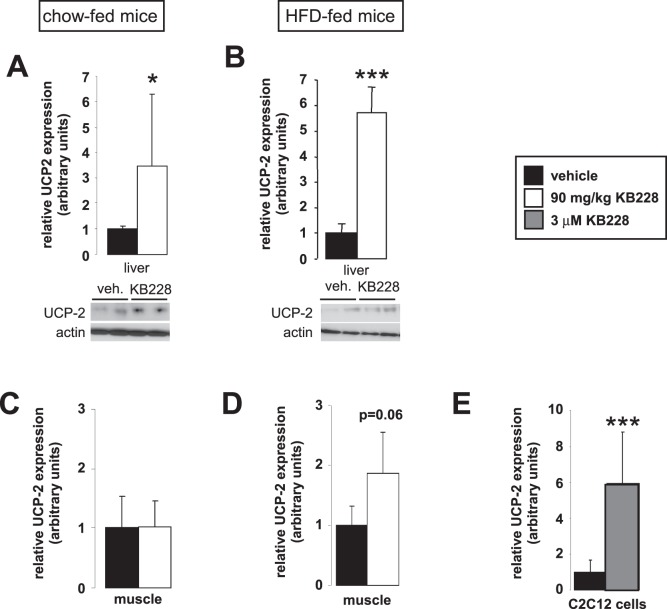
UCP2 induction by KB228. Chow-fed (n = 7/7, 6 months of age) and HFD-fed (n = 9/9, 6 months of age) C57/Bl6J male mice underwent vehicle or KB228 treatment. 2 hours post-treatment livers and gastrocnemius muscles were removed and homogenized. (A, B) In liver homogenates from chow-fed (A) or HFD-fed (B) mice UCP2 mRNA and protein levels were assayed in RT-qPCR reactions and Western blotting. (C, D) In skeletal muscle homogenates from chow-fed (C) or HFD-fed (D) mice UCP2 mRNA levels were measured in RT-qPCR reactions. (E) Differentiated C2C12 myoblasts were treated with 3 µM of KB228 for 8 hours then UCP2 expression was determined in RT-qPCR reactions. Error is given as SD on panel E. * and *** indicate statistically significant difference between vehicle and GPi-treated groups at p<0.05, or p<0.001, respectively.

Similarly to our findings in cellular models, KB228 induced UCP2 mRNA and protein content in the liver of chow and HFD-fed (diabetic) mice (Fig. 6A, B). However, the induction of UCP2 protein content was reduced in the diabetic mice when compared to the chow-fed control group. We assessed the expression of UCP2 in skeletal muscle samples. Although we did not detect changes in UCP2 expression in chow-fed mice (Fig. 6C), we have observed 2-fold induction in UCP2 mRNA levels in HFD-fed mice (Fig. 6D). Interestingly in C2C12 myoblasts a much larger, 6-fold enhancement of UCP2 expression was observed (Fig. 6E).

**Figure 7:**
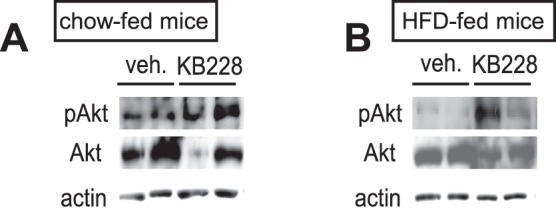
KB228 induced mTORC2 in mice. Chow-fed (n = 7/7, 6 months of age) and HFD-fed (n = 9/9, 6 months of age) C57/Bl6J male mice underwent vehicle or KB228 treatment (90 mg/kg). 2 hours post-treatment livers were removed and homogenized. From liver homogenates of chow-fed (A) and HFD-fed (B) mice Akt and phospho-Akt (^473^Ser) levels were determined by Western blotting.

To assess further metabolic rearrangements triggered by KB228, we examined the activity of certain protein kinases involved in energy homeostasis, such as mTORC2 (assessed by detecting phosphorylation of Akt2 on ^473^Ser) and Akt2 (assessed by detecting phosphorylation of GSK-3β on ^9/21^Ser) in mice. While we failed to detect changes in the phosphorylation of GSK-3β (data not shown), Akt2 phosphorylation on ^473^Ser was enhanced upon KB228 treatment in control and diabetic mice despite lower Akt2 protein content (Fig. 7A, B) highlighting marked activation of mTORC2.

## Discussion

In the present study we characterized the metabolic effects of a novel, potent GPi, KB228. As expected from previous studies with other GPi-s [8], [9], [15]–[18], [32], KB228 reduced serum glucose levels and increased hepatic glycogen content under both normoglycemic and insulin resistant, hyperglycemic conditions. Surprisingly, glucose clearance was primarily attributed to the liver. Prior studies have suggested the involvement of skeletal muscle in GPi-induced glucose clearance [8], however upon KB228 treatment we did not detect increased glucose uptake in skeletal muscle, suggesting that KB228 action was rather restricted to the liver. However, it must be noted that we have observed the induction of UCP2 in murine gastrocnemius muscle and C2C12 cells despite the lack of enhanced glucose uptake. Our data point to the involvement of skeletal muscle in the glucose oxidation providing a likely explanation for the phenotype observed by Baker and co-workers [8].

The potency of KB228 to reduce glucose levels and to influence downstream molecular events was reduced under hyperglycemic conditions. GP effectors act through several binding sites on the enzyme [33], [34]. KB228 displayed a mixed type inhibition suggesting the concurrent binding of the inhibitor to multiple sites, among them probably to the catalytic site. It is likely that the glucose moiety of KB228 competes with glucose for binding to the catalytic center of GP, therefore high glucose levels may reduce KB228 affinity to GP. Other glucose-based GPi-s were described to behave similarly under high glucose concentrations [9].

When we continued to uncover the molecular rearrangements and cellular effects induced by KB228 we found two parallel events that help the metabolic accommodation of cells to GPi treatment: induction of UCP2 expression and mTORC2 activity. UCP2 is a mitochondrial inner membrane protein that uncouples mitochondrial proton gradient from ATP production [35]. Higher level of uncoupling may induce the flux of the electron transport chain [36] therefore induction of UCP2 expression explains higher oxygen consumption. What could be the benefit from the induction of UCP2 expression? Excess glucose influx has been shown to produce hydroxyl radicals of mitochondrial origin due to the stalling of the mitochondrial electron transport chain [37]. UCP2 is capable of releasing that blockade therefore its induction is considered to protect against oxidative stress [38], [39]. It is presumable that UCP2 activation by KB228 treatment may have the same rationale, whereby UCP2 neutralize the excess glucose influx-induced mitochondrial free radical production analogously to the situation of enhanced hepatic fatty acid accumulation and catabolism [40], [41]. Furthermore, it is possible that UCP2 expression might have contributed to the glucose lowering effect of KB228 through enhancing energy expenditure in liver. In fact, long term GPi application was reported to have adverse effects characterized by hepatic lipid and glycogen deposition [42]. UCP2 overexpression, however may have beneficial effects over these adverse effects that could be exploited to counteract hepatic lipid and glycogen accumulation upon long term GPi treatment.

mTOR, which was also found to be induced by KB228 treatment, is a conserved Ser/Thr protein kinase functioning as a master regulator of metabolism existing in two distinct complexes called complex 1 (mTORC1) and complex 2 (mTORC2) [43]. The defining component of mTORC2 is Rictor (rapamycin-insensitive companion of mTOR) that may serve to recruit substrates to mTORC2. Little is known to date on the upstream inputs and functions of mTORC2, however it seems that mTORC2 is insensitive to nutrient levels, but it is activated in response to insulin and growth factor receptor activation [44]. Yet two targets of mTORC2 have been identified, Akt2 and SGK [44]. ^473^Ser phosphorylation of Akt2 seems to be an mTORC2 specific site. Phosphorylation of ^473^Ser Akt2 leads to a restricted-type activation of Akt2, whereby Akt2 can phosphorylate only a limited set of its downstream targets (such as FOXO1 and FOXO3) in contrast to phosphorylation and activation by mTORC1 [45]. In liver, mTORC2 activation induces nutrient storage (glycogen and fatty acid synthesis) and glycolysis [46], [47]. Therefore, mTORC2 activation may contribute to glycogen synthesis. Moreover, mTORC2 activity has been implicated in the hormonal rearrangement, termed hepatic satiety [47], a puzzling effect that could be investigated in relation to GP activity in the future.

The molecular events through which KB228 treatment leads to mTORC2 activation is unknown, but in any case, it is intriguing. That process can be termed retrograde signaling, as the inhibition of GP, an enzyme lying downstream of Akt2, in fact, leads to the activation of mTORC2 or alterations in insulin signaling, both lying upstream of Akt2. However, it cannot be excluded that simply glucose influx rearranges mTOR signaling, whereby cells sense glucose plenitude and turn towards storage that is glycogen build-up.

In summary, our data highlight that GP inhibition by KB228 treatment exerts more effects than a simple inhibition of catalytic activity. Our data show that glycogen metabolism interacts with mTORC2 and mitochondrial signaling. Recent research have shown that appropriate glycogen catabolism is a vital part of cellular glucose metabolism that is indispensable in sustaining cell cycle [48], [49] or preventing cellular senescence [49]. Taken together with the data presented hereby strongly suggests that GP might be involved in a complex metabolic regulatory network that could be exploited in the management of diabetes, metabolic diseases or anti-Warburg strategies.
